# Experimental scatter of the fatigue response of additively manufactured components: a statistical method based on the Profile Likelihood

**DOI:** 10.1038/s41598-023-40249-8

**Published:** 2023-09-15

**Authors:** A. Tridello, C. Boursier Niutta, M. Rossetto, F. Berto, D. S. Paolino

**Affiliations:** 1 Department of Mechanical and Aerospace Engineering, Politecnico di Torino, C.so Duca degli Abruzzi 24, 10129 Turin, Italy; 2 Department of Chemical Engineering, Materials and Environment, Università La Sapienza, 00185 Rome, Italy

**Keywords:** Mechanical properties, Mechanical engineering

## Abstract

The fatigue response of additively manufactured (AM) specimens is mainly driven by manufacturing defects, like pores and lack of fusion defects, which are mainly responsible for the large variability of fatigue data in the S–N plot. The analysis of the results of AM tests can be therefore complex: for example, the influence of a specific factor, e.g. the building direction, can be concealed by the experimental variability. Accordingly, appropriate statistical methodologies should be employed to safely and properly analyze the results of fatigue tests on AM specimens. In the present paper, a statistical methodology for the analysis of the AM fatigue test results is proposed. The approach is based on shifting the experimental failures to a reference number of cycles starting from the estimated P–S–N curves. The experimental variability of the fatigue strength at the reference number of cycles is also considered by estimating the profile likelihood function. This methodology has been validated with literature datasets and has proven its effectiveness in dealing with the experimental scatter typical of AM fatigue test results.

## Introduction

The research on the mechanical behaviour of additively manufactured (AMed) components is fundamental to guarantee their structural integrity and to further boost their diffusion. To this aim, the number of research activities investigating the quasi-static and fatigue response of AM parts has rapidly increased in the last few years. For what concerns the quasi-static mechanical properties, several experimental results have confirmed that the tensile strength is at least comparable to that of parts produced with traditional manufacturing processes, mainly due to the fine microstructure originating during the AM process^1–3^. On the other hand, the use of AM components in applications where cyclic loads are applied is currently limited and many concerns about their structural integrity under fatigue loads are still present^2,4–6^. Accordingly, components produced with traditional manufacturing processes are still preferred and considered safer, even if AM is a more sustainable and eco-friendly production process^7,8^, ensuring less material waste and allowing to produce lightweight components designed with topology optimization algorithms.

The research on the fatigue response of AM parts has been very active in the last few years. Several factors affect the fatigue response of AM parts and contribute to their low fatigue response, if compared to that of traditionally built parts. For example, residual stresses and the microstructure originating during the AM process have a significant role^9^. The design of appropriate heat treatments, however, can limit the influence of these factors^9^. On the other hand, manufacturing defects, like pores, lack of fusion defects, play the most important role^10–12^. Indeed, even if the defect size and density can be reduced and controlled through an optimization of the process parameters^13,14^, their random formation cannot be avoided and significantly affects the fatigue response. Moreover, defects induce local variation of the microstructure and the formation of regions with local severe plastic deformations, which can also affect the crack nucleation process and the following crack propagation, according to the recent literature. For example, an increment of the nitrogen content in the 316 L steel induce microstructural modifications which made the part more damage tolerant^15^. In Ref.^16^, the Authors showed that variations of the crystallographic orientation and microstructure around the defect originating the fatigue crack influence the fatigue response and contribute to the large experimental scatter typical of fatigue tests on AM specimens. Similarly, the plastic deformation around the critical defect induces mean stress relaxation which must be properly accounted for when dealing with the analysis of the fatigue response, for example with Finite Element Analyses (FEAs)^17^. According to this analysis, the defect, with its size and location, and the microstructure around it, with local modifications and local plastic deformations, strongly affect the fatigue response. Indeed, fatigue datasets of AM parts with failures originating from defects are characterized by large scatter and variability in the S–N plot, thus making it difficult to assess, for example, the influence of specific investigated factors or to define reliable design methodologies. Accordingly, one of the main scientific open issues is how to deal with this large and detrimental experimental scatter. Several methodologies have been therefore proposed to address it, investigating the factors that affect it, and modelling the influence of defects^12,18–22^. For example, in Refs.^23–25^, methodologies for the assessment of the fatigue response in function of the defect size, have been proposed starting from the Paris law and the Murakami model. The approaches in Refs.^26–28^, on the other hand, exploit the information on the defect population assessed with micro-CT analysis or statistics of Extreme Values and fracture mechanics methodologies to assess the fatigue response of AM parts. Derrick et al.^29^ proposed a model for estimating the P–S–N curves based on the specimen hardness, roughness and defect size, showing the variation of the fatigue curves with these factors. Similarly, Wits et al.^30^ focused on the variation of the slope of the S–N curve with the defect size, with the slope increasing as the defect size increases. In Refs.^12,18^, statistical methodologies to model the influence of the defect size on the fatigue life are developed. On the other hand, the influence of the AM process parameters on the defect population and the fatigue response is experimentally investigated through design of experiments methods in Refs.^13,14^. In Ref.^31^, a methodology for the design of AM parts based on artificial defects which control the crack nucleation process and reduce the experimental scatter is proposed and experimentally validated. This literature analysis on recently published papers proves the importance of accounting for the factors which mostly affect the fatigue response, i.e., the defects and the surrounding microstructure, to model the variability generally found in experimental tests. Furthermore, literature analysis has also shown that without the application of robust statistical methods validated on experimental data, unsafe or too conservative estimation of the fatigue strength can be obtained, with misleading interpretations of the experimental results and the influence of investigated factors. In Ref.^12^, the Authors have proposed an approach for the analysis of the results of tests on AM specimens, based on shifting the experimental failures to a reference number of cycles for the subsequent application of statistical methods. This method has been validated with literature datasets obtained through experimental tests on AM specimens, proving its validity.

In the present paper, the statistical methodology proposed in Ref.^12^ has been improved, accounting also for the uncertainty associated with the estimation of the quantiles of the P–S–N curves. The effective approach, based on shifting the experimental failures to a reference number of cycles, has been kept, but, instead of considering deterministic fatigue strengths at the reference number of cycles, their range of variability is modelled by exploiting the Profile Likelihood function properties. This improved methodology has been validated with literature datasets and has proven its capability and effectiveness, representing a reliable method for the analysis of AM test results.

## Methods

This Section deals with the proposed methodology and its implementation. In "Statistical methodology: analytical definition" the proposed methodology is described, whereas in "Implementation procedure". The implementation procedure is reported. For the sake of clarity, in the following the fatigue strength is defined as the value of stress amplitude at which a failure occurs after $Nf$ cycles, being ${N}_{f}$ the number of cycles to failure, whereas the fatigue limit is the value of stress amplitude corresponding to the asymptote of the S–N curve.

### Statistical methodology: analytical definition

Dealing with the large variability of the fatigue response of AM parts is a challenging scientific open issue. Indeed, the large experimental variability can conceal the influence of an investigated factor, if, for example, two fatigue datasets are compared, with misleading conclusions. Moreover, due to the high cost of AM specimens, the number of available data for a reliable estimation of the P–S–N curve is limited or, in general, smaller than that available for traditionally built specimens, thus further complicating the analysis. The most effective way to analyze AM datasets characterized by large scatter is to employ reliable and effective statistical methodologies. The procedure proposed in Ref.^12^ addresses this open problem and is briefly recalled in the following. The basic idea developed in Ref.^12^ is that failures can be shifted to a selected reference number of cycles to failure, ${N}_{ref}$, or to selected reference applied stress amplitude, ${s}_{ref}$, for subsequent statistical analyses, like the Analysis of Variance (ANOVA), hypothesis tests or analyses based on Montecarlo simulations^12^. The following steps are required for the application of this procedure:

1. The stress-life relationship, i.e., the P–S–N curves, are estimated from the experimental data. In Ref.^12^, the general model for the duplex P–S–N curves, i.e., the P–S–N curves covering the fatigue life range up to the Very High Cycle Fatigue (VHCF) life ranges, is employed. The model in Ref.^12^ accounts for the influence of defects with the so-called marginal P–S–N curves^32,33^, i.e., the P–S–N curves “averaged” by the defect size, which is assumed to follow a Largest Extreme Value Distribution (LEVD)^34^. Accordingly, for each failure, together with the number of cycles to failure and the applied stress amplitude, the defect at the origin of the fatigue failure should be reliably measured on the fracture surface.
2. Experimental failures are thereafter shifted at ${N}_{ref}$ or to ${s}_{ref}$. This operation is carried out by considering that each failure is crossed by a specific $\alpha$ quantile P–S–N curve. Following the estimated $\alpha$ quantile P–S–N curve, the shifted failure is obtained.
3. By repeating point two for each experimental failure, the experimental data are gathered together, and appropriate statistical methodologies can be applied, like the ANOVA analysis, whose use is prevented if the data are collected at different numbers of cycles to failure and applied stress amplitudes.

This methodology has been validated with AM literature data in Ref.^12^ and has proven its capability of comparing different AM datasets to assess the influence of the investigated factors in a reliable statistical framework. Despite its effectiveness, this methodology strongly depends on the initial estimation of the stress-life relationship, i.e., the parameters estimated by considering the experimental data should be as close as possible to the actual parameters (i.e., the parameters of the fatigue life population). Otherwise, the methodology may provide misleading results, especially if the number of available data is limited and the experimental variability is large. Due to the dependency between the estimated parameters and the experimental data (i.e., the estimated parameters are strongly dependent on the available sample, i.e., the set of experimental data), the fatigue strength at ${N}_{ref}$ is not a deterministic value but is, in turn, affected by the dataset variability and its numerosity. Therefore, an interval for the fatigue strength at ${N}_{ref}$ should be assessed, rather than a deterministic value.

The methodology proposed in the present paper addresses these weaknesses, thus modelling also the uncertainty associated with the fatigue strength at ${N}_{ref}$. A stress-life distribution with a linear decreasing trend and a final asymptote, i.e., the fatigue limit, has been considered. The influence of defects has not been directly modelled, making this method appropriate also for datasets for which the initial defect is not available. Equation (1) shows the cumulative distribution function (cdf), ${F}_{Y|x}$, of the random variable $Y={\mathrm{log}}_{10}\left({N}_{f}\right)$, conditioned to $x={\mathrm{log}}_{10}\left({s}_{a}\right)$, being ${s}_{a}$ the applied stress amplitude:

1 $${F}_{Y|x}=\Phi \left(\frac{y-\left(a+b\cdot x\right)}{{\sigma }_{Y}}\right)\Phi \left(\frac{x-{\mu }_{{X}_{l}}}{{\sigma }_{{X}_{l}}}\right),$$

being $\Phi \left(\cdot \right)$ the cdf of a standardized Normal distribution, $a$ and $b$ constant coefficients describing the linear decreasing trend in the finite life region, ${\mu }_{{X}_{l}}$ the mean of the fatigue limit distribution, ${\sigma }_{Y}$ and ${\sigma }_{{X}_{l}}$ the standard deviations of the fatigue life and of the fatigue limit distributions, respectively. The fatigue life and fatigue limit random variables are assumed to follow a Normal distribution. The set of unknown parameters in Eq. (1), ${\varvec{\theta}}=\left(a, b, {\mu }_{{X}_{l}},{\sigma }_{Y}, {\sigma }_{{X}_{l}}\right)$, are estimated from the experimentally available failures (being $n$ the number of failures) and runout specimens (being ${n}_{r}$ the number of runout specimens, if available) with the Maximum Likelihood Principle, i.e., by maximizing the Likelihood function $L\left[{\varvec{\theta}}\right]$ reported in Eq. (2):

2 $$L\left[{\varvec{\theta}}\right]={\prod }_{{i}_{f}=1}^{n}{f}_{Y|X=x}\left[{y}_{{i}_{f}};{x}_{{i}_{f}};\theta \right]\cdot {\prod }_{j=1}^{{n}_{r}}\left(1-{F}_{Y|X=x}\left[{y}^{*};{x}_{j};\theta \right]\right),$$

being ${f}_{Y|X=x}$ the probability density function (pdf) of the fatigue life distribution, ${y}_{{i}_{f}}$ and ${x}_{{i}_{f}}$ the logarithm of ${N}_{f}$ and ${s}_{a}$, respectively, of the ${i}_{f}$-th failure (${i}_{f}=1\dots n$), ${y}^{*}$ the logarithm of the runout number of cycles and ${x}_{j}$ the logarithm of ${s}_{a}$ of the $j$-th runout data ($j=1\dots {n}_{r}$). The ML estimate, $\widetilde{{\varvec{\theta}}}$, is the set of parameters that maximizes $L\left[{\varvec{\theta}}\right]$.

Following^12^, the estimated $\widetilde{{\varvec{\theta}}}$ are used to shift the data to the selected reference number of cycles to failure, ${N}_{ref}$: the $\alpha$ quantile S–N curves that cross the ${n}_{f}$ failures are computed from $\widetilde{{\varvec{\theta}}}$ and the corresponding fatigue strengths at ${N}_{ref}$ are then obtained. Differently from Ref.^12^, the Profile Likelihood function associated to the quantile of each fatigue strength shifted at ${N}_{ref}$, is computed. Accordingly, rather than a deterministic fatigue strength at ${N}_{ref}$, a statistical distribution of the quantile S–N curve is estimated. With this approach, the range of possible values of the shifted data is computed. Thereafter, random fatigue strengths can be extracted from the Profile Likelihood functions associated to each experimental failure to assess the empirical cumulative distribution function (ecdf) of the fatigue strength at ${N}_{ref}$. Alternatively, if two fatigue datasets must be compared, the ecdf of the difference between the random fatigue strengths at ${N}_{ref}$ can be computed to assess the statistical significance of the difference.

**Figure 1:**
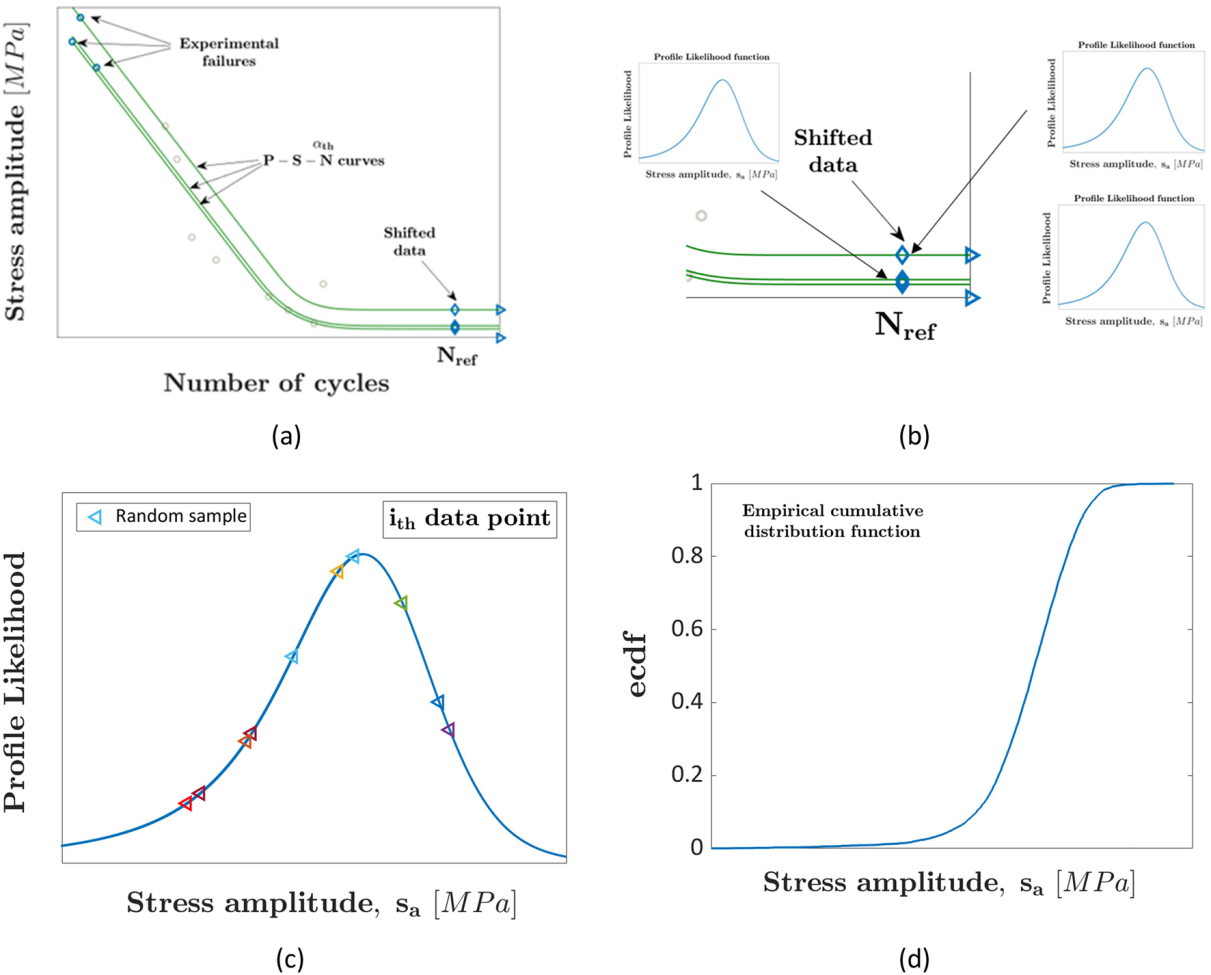
Proposed statistical method accounting for the uncertainty associated with the fatigue response of AM specimens: (**a**) procedure for shifting the experimental failures to the ${N}_{ref}$; (**b**) Profile Likelihood functions for the fatigue strengths shifted at ${N}_{ref}$; (**c**) random fatigue strength extraction by considering the estimated Profile Likelihood functions; (**d**) example of the empirical cumulative distribution function (ecdf) describing the fatigue strength distribution at ${N}_{ref}.$

Figure 1 helps clarifying the developed methodology: Fig. 1a shows the procedure for shifting the experimental failures once the parameters of the fatigue life distribution are estimated. Figure 1b shows examples of the Profile Likelihood functions estimated for the shifted experimental failures. Figure 1c shows the Profile Likelihood function with several randomly estimated fatigue strengths (denoted with triangle markers). Finally, Fig. 1d shows an example of ecdf of the fatigue strength at ${N}_{ref}$, estimated by considering the randomly extracted fatigue strengths.

It must be noted that the proposed approach focuses on shifting the experimental data at a specific number of cycles to failure. However, it can be also employed to assess the fatigue response in the fatigue life range of interest. Indeed, the first step of the proposed approach is the assessment of the P–S–N curves that provide the best fitting of the experimental data. The estimated P–S–N curves can be used to analyze the fatigue life of the investigated specimens, e.g., for design purposes. Alternatively, by assessing the fatigue strength at different ${N}_{ref}$ in the life range of interest, a design curve can be built point by point. Accordingly, the proposed method is not only limited to the analysis of the fatigue response at ${N}_{ref}$, but it can be extended to the analysis of the fatigue life and its variation with the number of cycles to failure.

### Implementation procedure

The methodology described in "Statistical methodology: analytical definition" allows for a reliable analysis and comparison of the fatigue response of components produced through AM process. The experimental scatter and the uncertainty associated with the estimation of a specific quantile of the P–S–N are moreover accounted for, overcoming the main weakness of the procedure proposed in Ref.^12^. However, this methodology requires a proper implementation which can be quite complex if not properly set, since, for example, repeated optimizations are necessary for the estimation of the Profile Likelihood function. Accordingly, the objective of this Section is to describe step by step the developed implementation procedure. The Matlab software has been used for the implementation.

**Figure 2:**
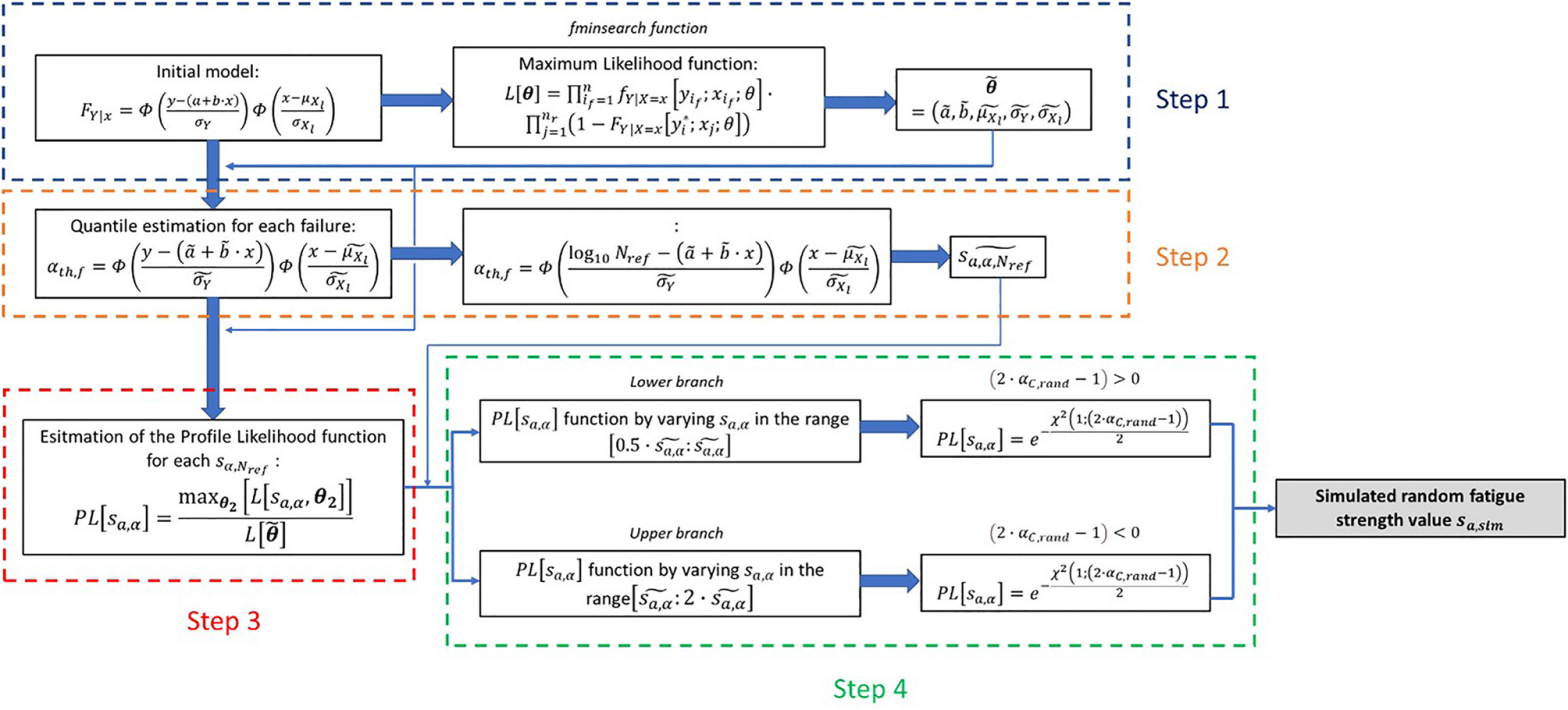
Flow chart of the developed procedure implemented in the Matlab code.

The flow chart in Fig. 2 shows the procedure implemented in the Matlab tool for the application of the above-described procedure.

The first step (Step 1 in Fig. 2) of the implemented procedure involves the estimation of the set of unknown parameters in the model in Eq. (1), ${\varvec{\theta}}=\left(a, b, {\mu }_{{X}_{l}},{\sigma }_{Y} {\sigma }_{{X}_{l}}\right)$. The Maximum Likelihood Principle is exploited for the parameter estimation, by using the optimization algorithm *fminsearch* implemented in Matlab, which is based on the *Nelder–Mead* simplex algorithm^35^. Thereafter (Step 2 in Fig. 2), the $\alpha$ quantile S–N curve associated to each experimental failure is obtained by solving Eq. (1) with the corresponding ${s}_{a}$ and ${N}_{f}$ values. Each failure is shifted to the selected ${N}_{ref}$ by solving Eq. (1) for the corresponding estimated $\alpha$ value and by considering $y={\mathrm{log}}_{10}\left({N}_{ref}\right)$.

The estimation of the Profile Likelihood function for each fatigue strength at ${N}_{ref}$ ($PL\left[{s}_{a,\alpha }\right]$, being ${s}_{a,\alpha }$ the $\alpha$ quantile associated to the fatigue strength at ${N}_{ref}$), which is the core of the methodology, requires, on the other hand, a more complex implementation (Step 3 in Fig. 2). The Profile Likelihood function can be written according to Eq. (3) as:

3 $$PL\left[{s}_{a,\alpha }\right]=\frac{{\mathrm{max}}_{{{\varvec{\theta}}}_{2}}\left[L\left[{s}_{a,\alpha },{{\varvec{\theta}}}_{2}\right]\right]}{L\left[\widetilde{{\varvec{\theta}}}\right]},$$

being ${{\varvec{\theta}}}_{2}=\left(a, b,{\sigma }_{Y}, {\sigma }_{{X}_{l}}\right)$ the set of the parameters of model and $L\left[\widetilde{{\varvec{\theta}}}\right]$ the Likelihood function computed for the ML estimate, $\widetilde{{\varvec{\theta}}}$. According to Refs.^36,37^, the $PL\left[{s}_{a,\alpha }\right]$ is a function of the $\alpha$ quantile of the fatigue strength (${s}_{a,\alpha }$). This can be achieved, according to the procedure described in Ref.^36^, by rearranging Eq. (1), i.e., by replacing ${F}_{Y|x}$ with $\alpha$ in Eq. (1) to obtain an expression of ${\mu }_{{X}_{l}}$ as a function of ${s}_{a,\alpha }$, i.e., ${\mu }_{{X}_{l}}={\mu }_{{X}_{l}}\left({s}_{a,\alpha }\right)$:

4 $${\mu }_{{X}_{l}}\left({s}_{a,\alpha }\right)={s}_{a,\alpha }-{\Phi }^{-1}\left(\frac{\alpha }{\Phi \left(\frac{\mathrm{ln}\left({N}_{f,\alpha }\right)-\left(a+b\cdot \mathrm{ln}\left({s}_{a,\alpha }\right)\right)}{{\sigma }_{Y}}\right)}\right)\cdot {\sigma }_{{X}_{l}},$$

By replacing Eq. (4) in Eqs. (1–3), the cdf and the pdf of the fatigue life distribution and the Profile Likelihood as a function of ${s}_{a,\alpha }$ are obtained. If a monotonic trend without a fatigue limit allows for a better fitting of the experimental data, the same procedure can be followed, e.g., by setting the cdf of the fatigue limit equal to 1 in Eq. (1) and by obtaining from this expression one of the unknown parameters, e.g., $a$, as a function of ${s}_{a,\alpha }$.

The $PL\left[{s}_{a,\alpha }\right]$ trend is then obtained by finding the set of parameters ${{\varvec{\theta}}}_{2}$ that maximizes $L\left[{s}_{a,\alpha },{{\varvec{\theta}}}_{2}\right]$. The ${s}_{a,\alpha }$ values in $L\left[{s}_{a,\alpha },{{\varvec{\theta}}}_{2}\right]$ are varied in a range close to the $\widetilde{{s}_{a,\alpha }}$, i.e., the quantile of the fatigue strength estimated by considering the set of parameters $\widetilde{{\varvec{\theta}}}$. Differently from Ref.^36^, where this procedure has been followed to estimate only the lower bound of the fatigue strength quantile, in this work the upper bound of the $PL\left[{s}_{a,\alpha }\right]$ is also assessed. Accordingly, the following steps are applied (Step 4 in Fig. 2):

1. The $PL\left[{s}_{a,\alpha }\right]$ for $\widetilde{{s}_{a,\alpha ,{N}_{ref}}}$, i.e., the fatigue strength of the data shifted at ${N}_{ref}$, is at first computed and must be equal to 1.
2. The $PL\left[{s}_{a,\alpha }\right]$ is then computed by varying ${s}_{a,\alpha }$ till $PL\left[{s}_{a,\alpha }\right]$ falls below the $2{e}^{-2}$ lower threshold value, with steps adjusted depending on the datasets. The maximization of $L\left[{s}_{a,\alpha },{{\varvec{\theta}}}_{2}\right]$ is carried out with the *fminsearch* function implemented in Matlab. With this procedure the lower branch of the $PL\left[{s}_{a,\alpha }\right]$ is computed and a set of discrete $PL\left[{s}_{a,\alpha }\right]$ points for each considered ${s}_{a,\alpha }$ in the investigated range is obtained. To obtain a continuous function, which will be useful in the following steps, an interpolation with the Piecewise Cubic Hermite Interpolating Polynomial (PCHIP) is carried out. The PCHIP is chosen for its fitting capability and since less computationally expensive with respect to other interpolation methods implemented in Matlab.
3. The procedure described at point 2 is repeated to estimate the upper branch of the $PL\left[{s}_{a,\alpha }\right]$ function. The same steps are repeated, but, in this case, ${s}_{a,\alpha }$ is varied between ${s}_{a,\alpha ,{N}_{ref}}$ and the value of ${s}_{a,\alpha }$ providing $PL\left[{s}_{a,\alpha }\right]$ equal to $2{e}^{-2}$. At the end of this step, the $PL\left[{s}_{a,\alpha }\right]$ and, accordingly, the Profile Likelihood function for the investigated failure shifted at ${N}_{ref}$ is computed. This procedure is repeated for each failure within the dataset.

The final step of the proposed procedure involves the extraction of random fatigue strengths from the estimated Profile Likelihood functions of the fatigue failures at ${N}_{ref}$. Firstly, random probabilities, ${\alpha }_{C, rand}$ are simulated by considering a uniform distribution with the *rand* function in Matlab. For each experimental data shifted at ${N}_{ref}$, ${n}_{sim}=1000$ probabilities are simulated. According to Ref.^12^, for each simulated ${\alpha }_{C, rand}$ value, the corresponding random fatigue strength (${s}_{a,sim}$) is obtained starting from Eq. (5):

5 $$PL\left[{s}_{a,\alpha }\right]=\frac{{\mathrm{max}}_{{{\varvec{\theta}}}_{2}}\left[L\left[{s}_{a,\alpha },{{\varvec{\theta}}}_{2}\right]\right]}{L\left[\widetilde{{\varvec{\theta}}}\right]}\ge {e}^{-\frac{{\chi }^{2}\left(1;1-{\beta }_{th}\right)}{2}},$$

where ${\chi }^{2}\left(1;1-{\beta }_{th}\right)$ is the $\left(1-{\beta }_{th}\right)$-th quantile of a Chi-square distribution with 1 degree of freedom. In general, the ${s}_{a,\alpha }$ values satisfying Eq. (5) are the $\left(1-{\beta }_{th}\right)\%$ lower and the upper confidence bounds for ${s}_{a,\alpha }$. In this work, the one side confidence interval has been considered for estimating the random fatigue strength ${s}_{a,sim}$ corresponding to ${\alpha }_{C, rand}$, i.e., $\left(1-{\beta }_{th}\right)$ in Eq. (5) corresponds to $\left(2\cdot {\alpha }_{C, rand}-1\right)$. In particular, ${s}_{a,sim}$ is computed according to the following conditions:

- If $\left(2\cdot {\alpha }_{C, rand}-1\right)>0$, the lower branch of the $PL\left[{s}_{a,\alpha }\right]$ is considered and ${s}_{a,sim}$ is estimated by solving Eq. (5) with $\left(1-{\beta }_{th}\right)=\left(2\cdot {\alpha }_{C, rand}-1\right)$.
- If $\left(2\cdot {\alpha }_{C, rand}-1\right)<0$, the upper branch of the $PL\left[{s}_{a,\alpha }\right]$ is considered and ${s}_{a,sim}$ is estimated by solving Eq. (5) with $\left(1-{\beta }_{th}\right)=\left(2\cdot {\alpha }_{C, rand}-1\right)$.

Practically, ${s}_{a,sim}$ is the value which minimizes the difference between the estimated PCHIP interpolating function and ${e}^{-\frac{{\chi }^{2}\left(1;\left(2\cdot {\alpha }_{C, rand}-1\right)\right)}{2}}$. By repeating this procedure for the $n$ experimental failures, $n\cdot {n}_{sim}$ values of ${s}_{a,sim}$ are obtained. The ecdf of the fatigue strength at ${N}_{ref}$ can be finally computed (an example is shown in Fig. 1d) and appropriate statistical methodologies can be employed, as detailed in "Validation with literature data".

## Validation with literature data

In this section, the proposed methodology is validated with literature datasets. The objective is to verify the applicability of the methodology, whose results are also compared with those obtained in Ref.^12^. The experimental data have been digitized with the software *Engauge* from the original paper, if not available in tabular form.

In "Ti6Al4V literature data: influence of AM process and HIP up to the VHCF regime" and "Ti6Al4V literature data: influence of AM process and post-treatments in the High Cycle Fatigue regime", the methodology is validated with the Ti6Al4V data obtained in^11,38^, respectively. In "T6Al4V literature data: influence of the building orientation" the datasets obtained through tests on 23 ELI Ti6Al4V are analyzed^39^. In "AlSi10Mg literature data: effect of the hatch spacing and building orientation", the data obtained by testing AlSi10Mg alloy specimens in Ref.^40^ are considered, whereas the test results in Ref.^41^ on a maraging steel are finally considered in "Maraging steel".

For the sake of clarity, Z building orientation and XY building orientation refer to the building orientation with the specimen axis perpendicular and parallel to the building platform, respectively. Moreover, the ecdfs have been estimated in the following with the Kaplan–Meier estimator implemented in the Matlab software. In the interval plot reported in the following analysis, the blue interval plot is the one estimated with the procedure developed in the present paper (indicated with “PL”), whereas the grey interval plot is the one estimated with the deterministic approach developed in reference^12^ (indicated with^12^).

The validation on datasets obtained in the literature by testing AM parts made with different materials and by varying the process parameters (e.g., the building orientation, the hatch spacing) or the post-treatment (polishing or as built surface) confirms and proves the effectiveness and the strengths of the proposed general approach, not requiring complex analyses of the fracture surfaces to assess the defect originating failure and being general and not limited to a particular investigated failure mode or specific testing condition or material.

### Ti6Al4V literature data: influence of AM process and HIP up to the VHCF regime

In this Section, the developed methodology is validated with the Ti6Al4V alloy data in Ref.^38^. In Ref.^38^, experimental tests on Ti6Al4V specimens produced with different AM processes have been carried out to investigate the influence of the manufacturing process, Selective Laser Melting (SLM) and Electron Beam Melting process (EBM), and of the HIP (Hot Isostatic Pressing) process on the fatigue response up to $5\times {10}^{9}$ cycles (in the VHCF region). The SLM specimens have been tested in two conditions: after a stress relief heat treatment, with a heating temperature of 800 °C, and after the HIP process (920 °C for 2 h at 1000 bar in an argon atmosphere). Fully reversed ultrasonic fatigue tests (loading frequency of 20 kHz) have been carried out on specimens built in Z direction and obtained through a machining process starting from bars produced with the investigated SLM and the EBM processes. In the following, according to Ref.^38^, “SLM-1B” specimens are the specimens produced with the SLM process and subjected to the stress relief heat treatment, “SLM-2” specimens are the specimens produced with the SLM process and subjected to the HIP process, “EBM” are the specimens produced with the EBM process. In the original paper^38^, the Authors concluded that the best fatigue performance can be achieved with the SLM process followed by the HIP process (SLM-2 batch), which allows to close pores and to reduce their size, besides relieving residual stresses. On the other hand, the SLM-1B and EBM fatigue responses have been found to be quite similar. The specimens produced with the EBM, however, have not required a stress relief heat treatment stress since produced with a heated building chamber. These conclusions have been drawn by the Authors by analyzing in detail the defects at the origin of the fatigue failures, but without a statistical analysis of the stress-life relationship.

**Figure 3:**
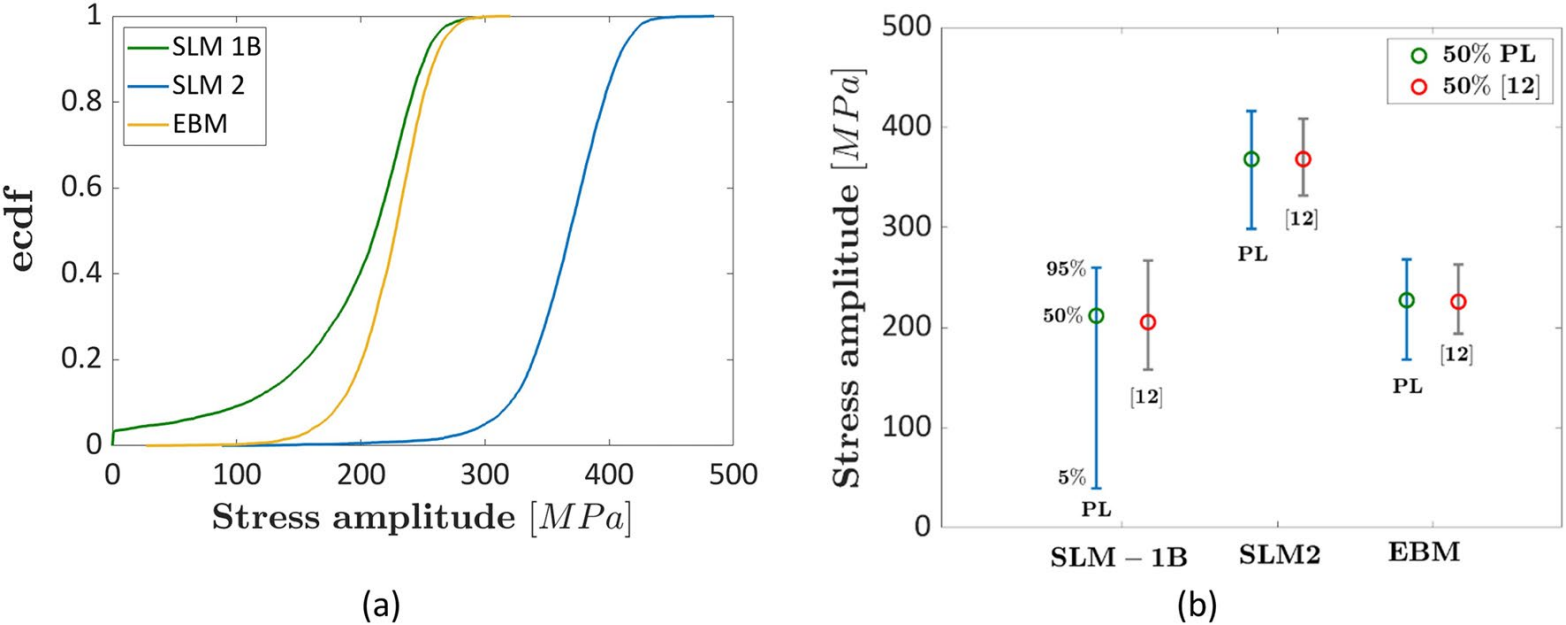
Experimental results in Ref.^38^: (**a**) ecdf of the fatigue strengths at ${N}_{ref}={3\times 10}^{9}$; (**b**) interval plot of the fatigue strength showing the 5%, 50% and 95% quantiles.

Figure 3 shows the results obtained with the proposed methodology. A linear model without a fatigue limit has been considered, according to the experimental trend. Figure 3a plots the ecdfs of the fatigue life at ${N}_{ref}={3\times 10}^{9}$ cycles, whereas the interval plots obtained from the ecdfs are shown in Fig. 3b, together with the interval plots estimated with the deterministic approach in Ref.^12^ The 5%, 50% and 95% quantiles are shown in Fig. 3b.

According to Fig. 3, the conclusions drawn by the Authors in Ref.^38^ are confirmed by the proposed statistical methodology, i.e., the “SLM-1B” and the “EBM” specimens are characterized by the same fatigue response (equal to about 245 MPa in the original paper by considering the interpolating line), whereas the HIP treatment strongly improves the fatigue response with the SLM-2 specimens showing the best performance (equal to about 390 MPa in the original paper). The interval plots in Fig. 3b for the SLM-1B and the EBM data overlap, whereas they are both below the interval plot for the SLM-2 batch. Similar results and the same trend have been obtained by applying the methodology in Ref.^12^. However, the intervals estimated in Ref.^12^ with the deterministic approach are significantly smaller, since the uncertainty associated with the fatigue strength at ${N}_{ref}$, neglected in Ref.^12^, is here considered. On the other hand, the median fatigue strength is close, with limited differences.

**Figure 4:**
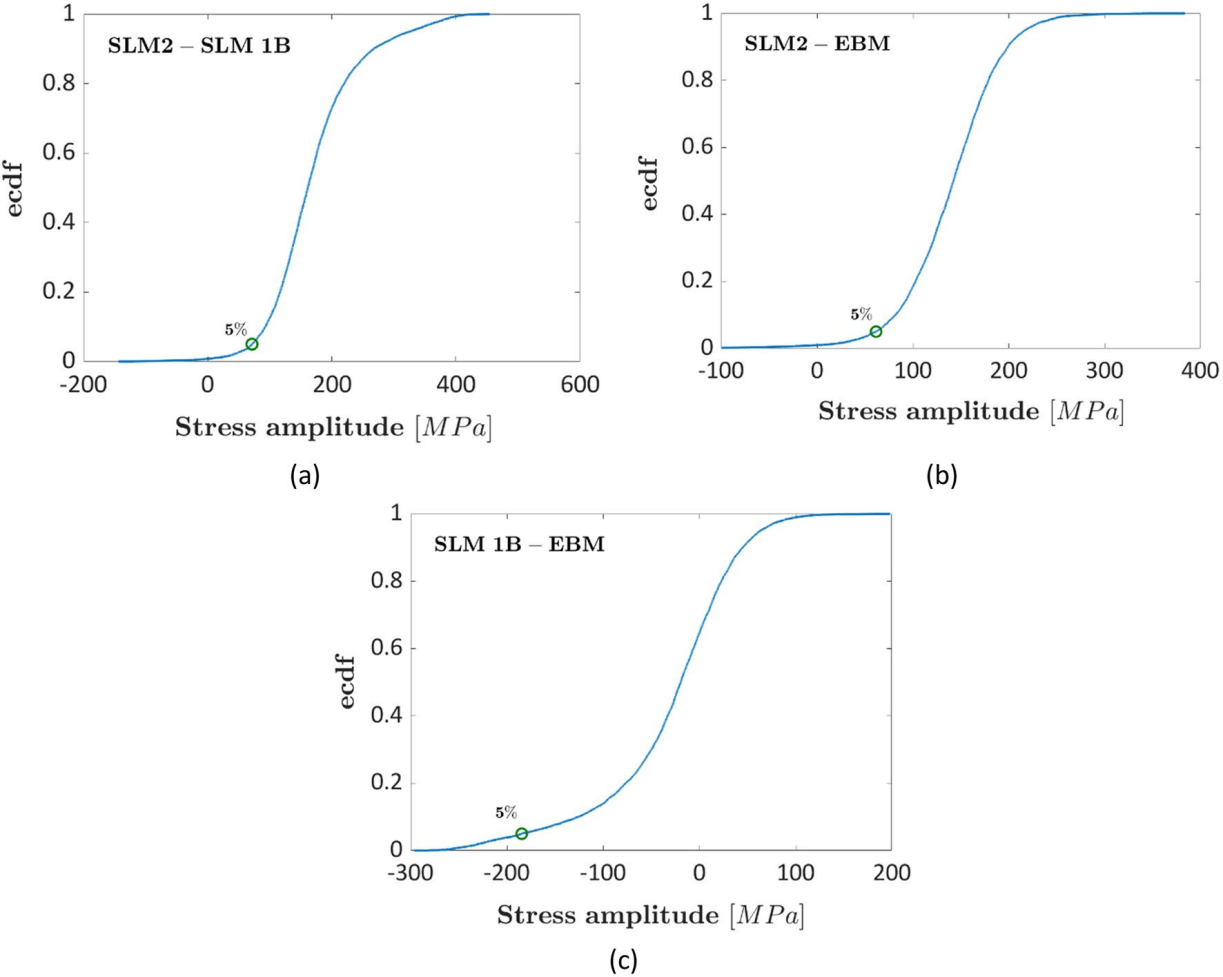
Experimental results in Ref.^38^, ecdf of the differences between the fatigue strengths: (**a**) SLM-1B and SLM-2; (**b**) SLM2 and EBM; (**c**) SLM1B and EBM.

Figure 4a plots the ecdf of the difference between the fatigue strengths of SLM-2 and SLM-1B datasets, Fig. 4b plots the ecdf of the difference between the fatigue strengths of SLM-2 and EBM datasets and Fig. 4c plots the ecdf of the difference between the fatigue strengths of SLM-1B and EBM datasets. The green marker corresponds to the 5% significance level.

Figure 4 further confirms the results of the analysis carried out in Fig. 3, with the difference between the SLM1B and the SLM2 and between the SLM2 and the EBM fatigue strengths being statistically significant, since the probability associated with the zero difference is below a 5% significance level. On the other hand, it is not possible to highlight a significant difference between the fatigue response of EBM and SLM-1B datasets, with the zero difference largely above the considered 5% significance level.

### Ti6Al4V literature data: influence of AM process and post-treatments in the high cycle fatigue regime

In Ref.^11^, the influence of the manufacturing process and post-treatments on the fatigue response up to the high cycle fatigue (HCF) life region is investigated. Rotating bending fatigue tests have been carried out up to failure or up to 10^7^ cycles tests on dogbone specimens produced with the Direct Metal Laser Sintering (DMLS) and the EBM processes along the Z direction. Before the fatigue tests, the specimens have been mechanically polished with fine-grain sandpaper (#600 emery paper). The building chamber has been preheated during the EBM process, whereas a stress relief heat treatment has been applied after the DMLS manufacturing process to relieve residual stresses. An approximate fatigue limit has been computed in the paper by considering the runout specimens in the S–N plot, being in the range [240–260] MPa for the EBM specimens and close to 370 MPa for the DMLS process.

**Figure 5:**
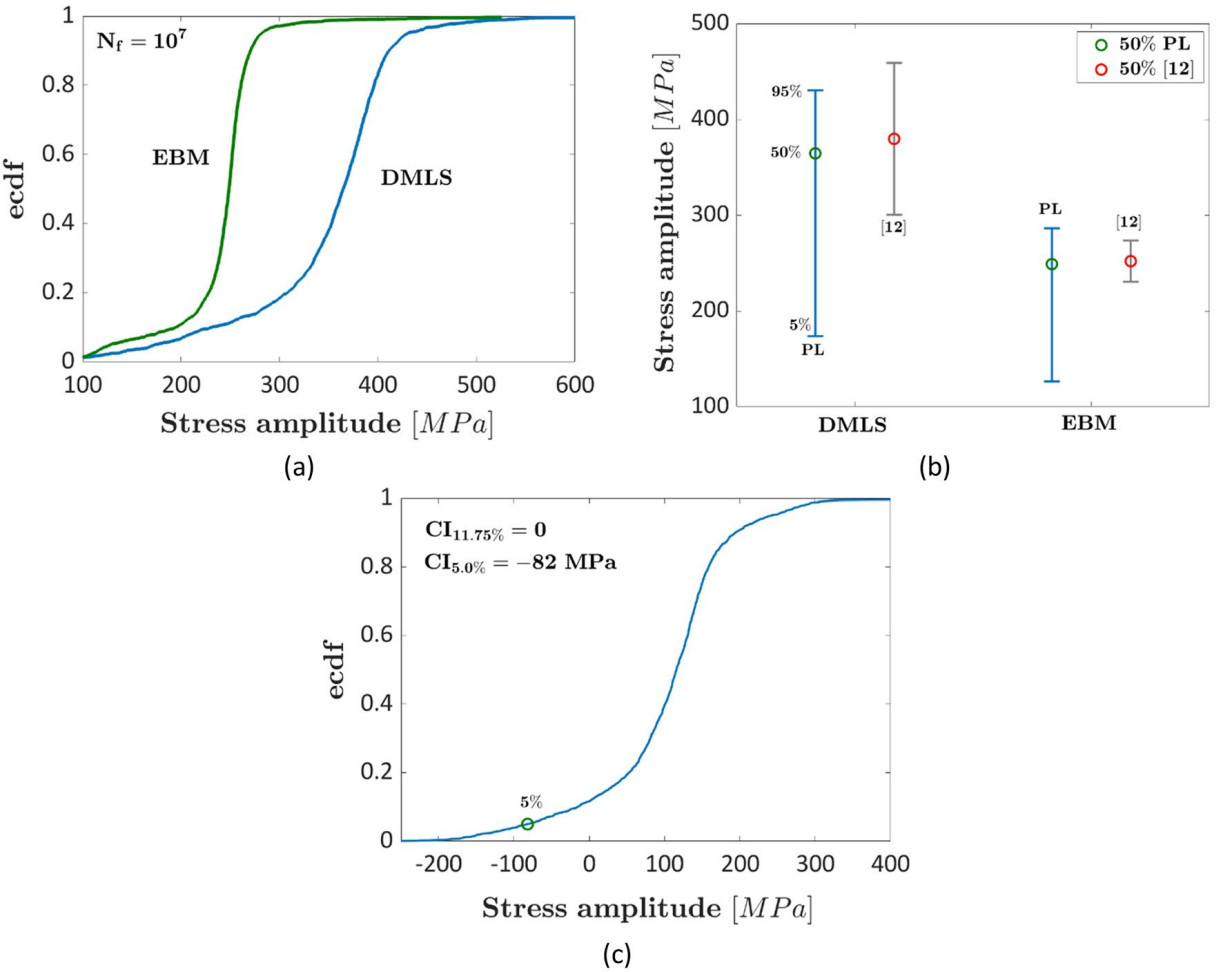
Experimental results in Ref.^11^ for polished specimens produced through DMLS and EBM processes at ${N}_{ref}={10}^{7}$: (**a**) ecdf of the fatigue response; (**b**) interval plot of the fatigue strength; (**c**) ecdf of the difference of the fatigue strengths.

Figure 5 shows the results of the analyses on the datasets in Ref.^11^ at ${N}_{ref}={10}^{7}$. A linear model with fatigue limit has been considered for the analysis. Figure 5a shows the ecdf of the fatigue strength, Fig. 5b shows the interval plot (with the 5% and the 95% quantiles) and Fig. 5c shows the ecdf of the difference of the fatigue strengths at ${N}_{ref}$. In Fig. 5b the interval plot estimated with the deterministic approach in Ref.^12^ are also reported.

According to Fig. 5, the median fatigue strengths are equal to 364 MPa and 250 MPa for the DMLS and the EBM datasets, respectively. The estimated fatigue strengths are close to the approximated value estimated by the authors and in Ref.^12^. The DMLS dataset shows a larger fatigue strength, as can be seen in Fig. 5b, but the experimental scatter is wide, providing wide intervals for this specific production process. The main reason can be attributed to the large uncertainty and variability of the fatigue response in the HCF life range, with less available data for a reliable estimation. In agreement with the analyses in "Ti6Al4V literature data: influence of AM process and HIP up to the VHCF regime", the intervals estimated with the deterministic approach in Ref.^12^ are larger, as expected. The importance of accounting for this uncertainty is more evident in Fig. 5c, where the ecdf of the difference between the fatigue strengths is plotted. Indeed, even if the median fatigue strength of DMLS specimens is larger, the difference cannot be considered significant with a significance level up to about 10%. On the other hand, in Ref.^12^ the difference was statistically significant for a significance level close to 5%. These analyses suggest that the proposed method, which accounts for the uncertainty of the fatigue strength at ${N}_{ref}$, provides more conservative results and uncertainty intervals, pointing out the need of increasing the amount of data in that specific investigated life range.

**Figure 6:**
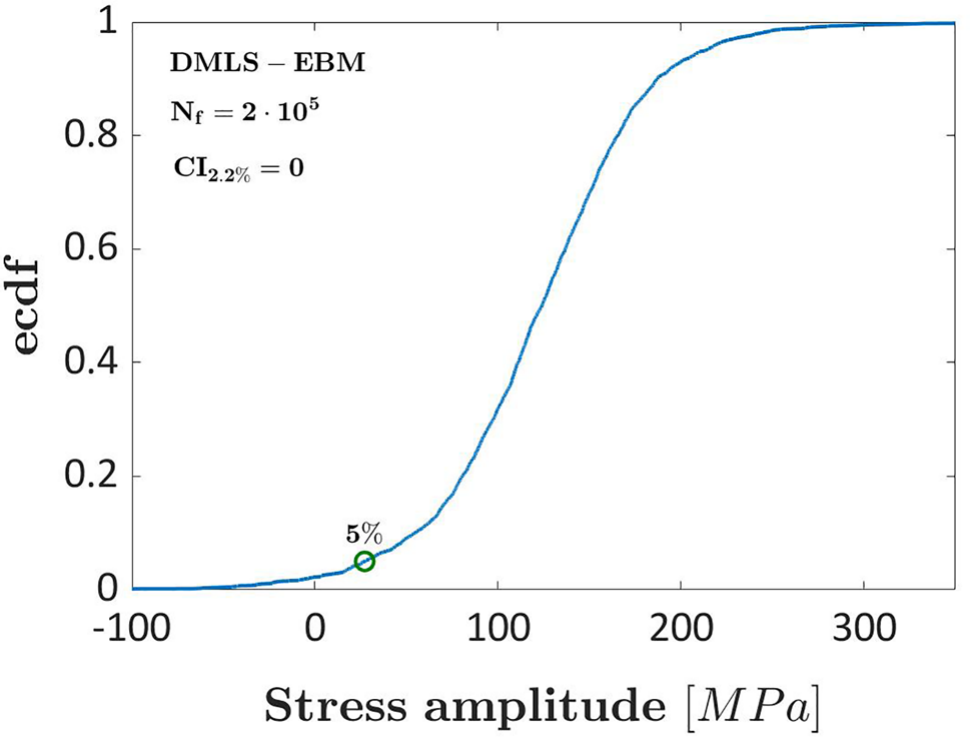
Ecdf of the difference of the fatigue strength for the experimental results in Ref.^11^ for polished specimens produced through DMLS and EBM processes at ${N}_{ref}=2{\times 10}^{5}$ cycles.

The same analysis has been carried out at ${N}_{ref}=2{\times 10}^{5}$ cycles, in the finite fatigue life region, where more data are available to estimate the fatigue strength and its variability. The ecdf of the difference between the fatigue strengths of DMLS and EBM specimens is shown in Fig. 6.

According to Fig. 6, the difference of the fatigue strengths at ${N}_{ref}=2{\times 10}^{5}$ cycles has become statistically significant up to a 2.2% significance level. This result confirms that different conclusions can be drawn depending on the number of data available in a specific life range and the importance of accounting for the experimental variability. Without considering the experimental variability, these conclusions cannot be drawn. From another point of view, this result may also suggest that the influence of a specific factor can vary with the fatigue life, i.e., it can have a larger influence depending on the number of cycles to failure, due to the variation in the experimental variability of the dataset.

The methodology proposed in the present paper can be applied even if the defect originating failure is not available, differently from Ref.^12^. This is the case, for example, of fatigue failures originating from the specimen surface due to high surface roughness, e.g., in as-built specimens. In Ref.^11^, the influence of the HIP process in as-built specimens manufactured through the SLM and the EBM process has been experimentally investigated. The specimens are characterized by similar hardness (369 HV for the EBM and 378 HV for the DMLS) and by different surface roughness (Ra in the range [32–42] µm for the EBM specimens and in the range [10–13] µm for the DMLS specimens). During the HIP process, the specimens have been kept at 920 °C and 100 MPa for 2 h and thereafter cooled in the furnace. All the fatigue failures have originated from surface defects whose characteristic size has not been measured. The approximate fatigue limits, i.e., the fatigue limits estimated by the Authors by considering the runout specimens, have been estimated to be equal to 140 MPa for the EBM specimens and equal to 155 MPa for the DMLS specimens. For the application of the proposed methodology, a linear decreasing trend for the DMLS and the EBM datasets has been considered. Indeed, a model with a fatigue limit has been also considered, but the available failures and runout data are limited and do not allow for a reliable estimation of an asymptotic trend. Accordingly, a monotonic decreasing trend is more appropriate for fitting the data.

**Figure 7:**
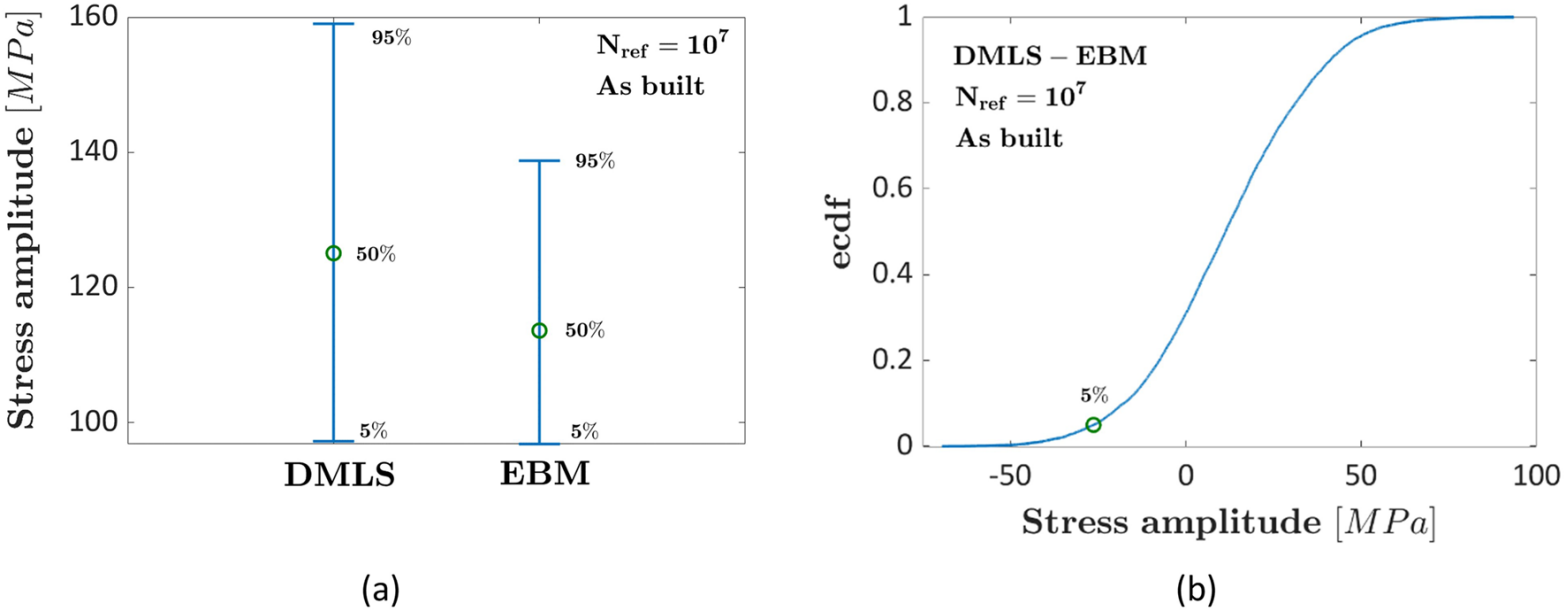
Experimental results in Ref.^11^ for as-built specimens subjected to the HIP process: (**a**) interval plot of the fatigue strength at ${N}_{ref}={10}^{7}$ cycles; (**b**) ecdf of the difference of the fatigue strength at ${N}_{ref}={10}^{7}$ cycles.

Figure 7a compares the interval plot (5% and 95% quantiles are shown) for the as-built DMLS and the EBM datasets at ${N}_{ref}={10}^{7}$ cycles, whereas in Fig. 7b the ecdf of the difference of the fatigue strengths for the same ${N}_{ref}={10}^{7}$ is shown.

According to Fig. 7a, the estimated median fatigue strength at ${N}_{ref}={10}^{7}$ cycles is smaller than that estimated in the original paper for both AM processes, being equal to 120 MPa and 110 MPa for the DMLS and the EBM datasets, respectively. The main reason can be attributed to the approach followed for assessing the fatigue strength at ${N}_{ref}={10}^{7}$: in Ref.^11^, an approximated fatigue limit is estimated by considering the runout data, whereas in this paper a methodology based on the stress-life relationship model that best fits the experimental data and accounts for the randomness associated with the fatigue strength at ${N}_{ref}$ has been employed. Accordingly, the estimated fatigue strength tends to be more conservative. However, the trend found with the proposed method is the same in Ref.^11^, with the median DMLS fatigue strength being above the EBM fatigue strength. The difference is, however, limited and, according to Fig. 7b, cannot be considered statistically significant. Therefore, it can be concluded that the HIP process has no effect on the fatigue response of as-built DMLS and EBM specimens, confirming moreover that the proposed method can be reliably employed even if the initial defect size is not available.

### T6Al4V literature data: influence of the building orientation

The influence of the building orientation has been also investigated in Ref.^39^ by testing a grade 23 ELI Ti6Al4V alloy up to ${N}_{f}={2\times 10}^{6}$ cycles. Experimental tests have been carried out at a stress ratio of 0.1 on machined specimens produced through an SLM process and after heat treatment (annealing at 850 °C for 2 h and furnace cooling). The effect of the building orientation has been investigated by testing specimens produced in the XY and Z directions and at 45°. For the XY and Z directions, two batches have been tested, whereas for the 45° building orientation, three batches have been tested. A large variability has been found between the tested batches for each building orientation. This has been attributed to the different defect sizes and demonstrates the large variability of AM fatigue results, even for the same set of process parameters.

**Figure 8:**
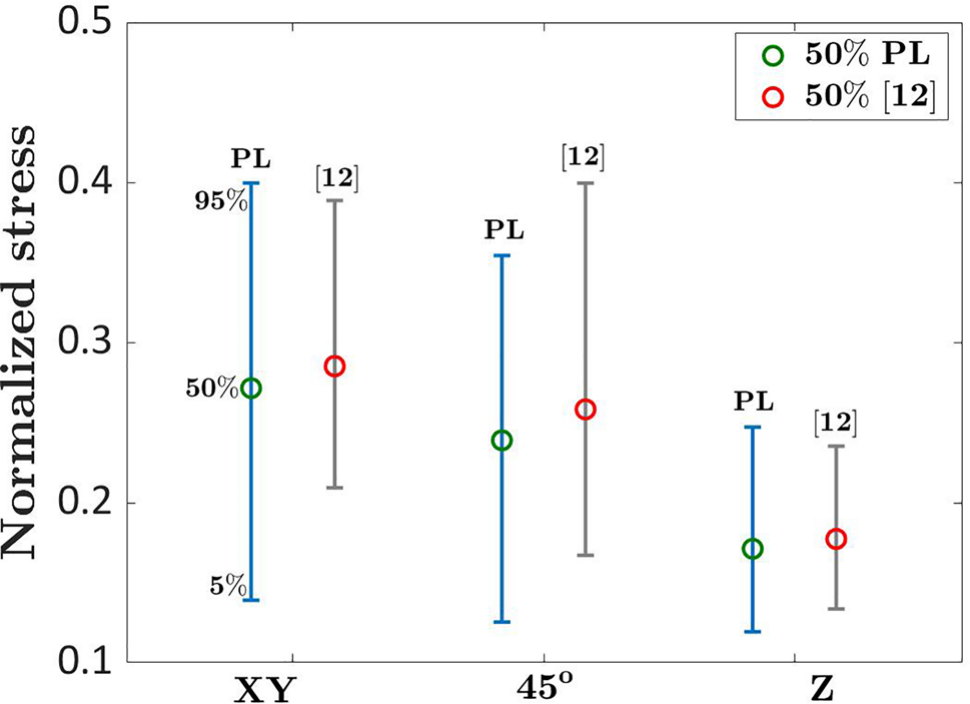
Experimental results in Ref.^39^ on grade 23 ELI Ti6Al4V to investigate the influence of the building orientation at ${N}_{ref}={10}^{7}$ cycles.

Figure 8 compares the interval plots (5% and 95% quantiles) of the normalized fatigue strengths estimated for the investigated building orientations at ${N}_{ref}={10}^{7}$ cycles, together with the intervals estimated with the deterministic approach in Ref.^12^ For each building orientation, all the data have been considered together, without distinguishing between the batches. In the original paper, the horizontal asymptote of the S–N curve for the investigated batches has been found in the range [0.45–0.55] for the XY building orientation, [0.40–0.60] for the 45° building orientation and [0.27–0.40] for the Z building orientation.

Figure 8 confirms the trend of the fatigue strength found in Ref.^12^, with the median fatigue strength decreasing from the XY to the Z building orientation (XY with the highest fatigue strength and Z with the smallest fatigue strength). The median fatigue strength is conservatively smaller than the one found in Ref.^12^, but the difference is limited. As in Ref.^12^, the XY and the 45° building orientations are characterized by a larger scatter, whereas the Z building orientation shows the smallest interval. The large estimated variability, however, does not allow to draw statistically significant conclusions on the influence of the building orientation on the fatigue response of the investigated Ti alloy, even if a decreasing trend for the median fatigue strength is clear. Accordingly, it can be concluded that the fatigue strength decreases with the building orientation, but this effect is concealed by the large experimental scatter. The intervals estimated with the deterministic approach in Ref.^12^ follow the same trend found with the methodology developed in the present paper (i.e., larger for the XY and the 45° building directions), but they are smaller, as expected.

### AlSi10Mg literature data: effect of the hatch spacing and building orientation

**Figure 9:**
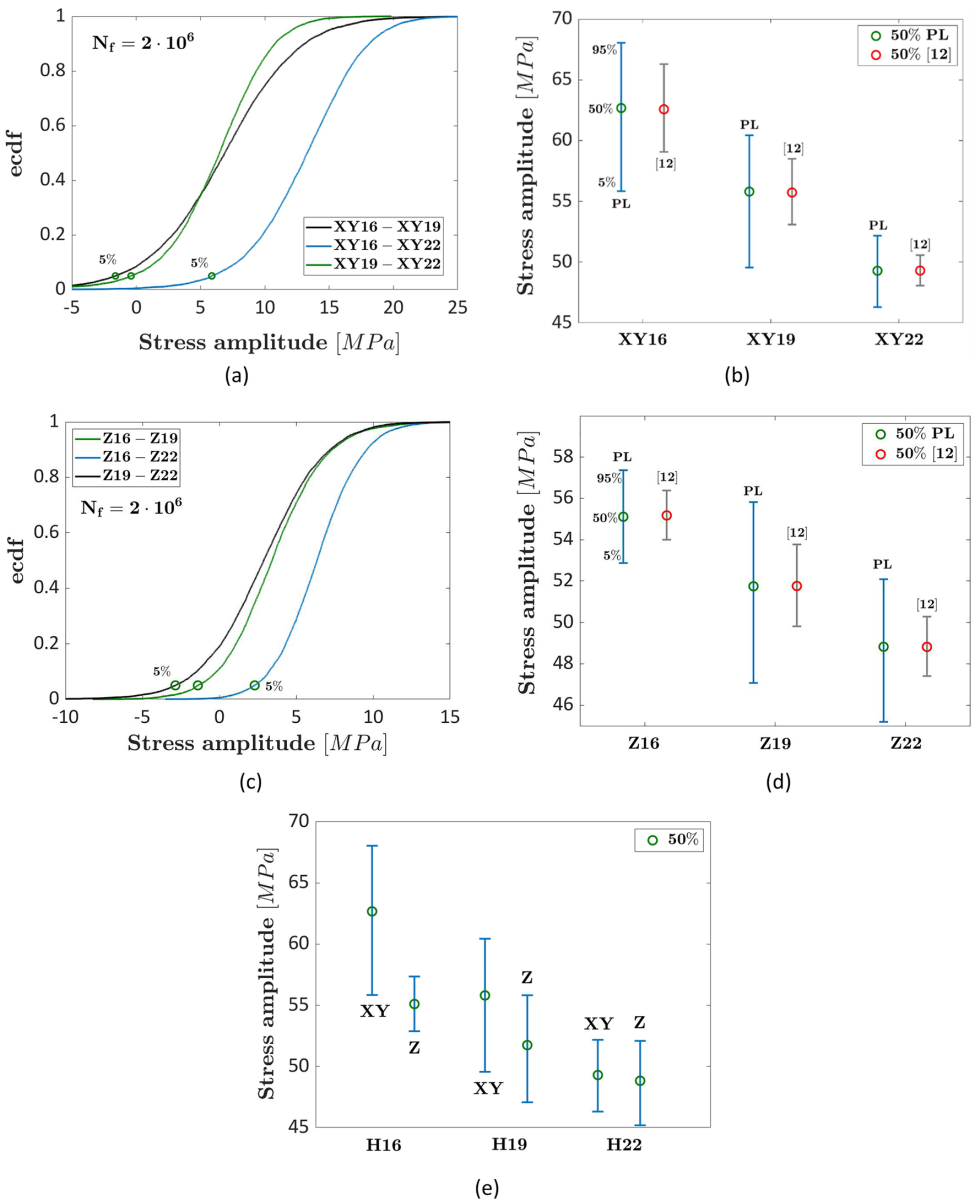
Experimental results in Ref.^40^ on AlSi10Mg specimens at ${N}_{ref}={2\times 10}^{6}$ cycles: (**a**) ecdf of the differences between the fatigue strength for the XY building direction; (**b**) interval plot of the fatigue strength for the XY building direction; (**c**) ecdf of the differences between the fatigue strength for the Z building direction; (**d**) interval plot of the fatigue strength for the Z building direction; (**e**) interval plot of the fatigue strengths for the investigated hatch spacings and building directions.

In Ref.^40^, the influence of the building orientation and the hatch spacing on the fatigue response of AlSi10Mg specimens has been investigated. Experimental tests at a stress ratio of 0.1 have been carried out at 80 MPa and 100 MPa (stress amplitude) on heat-treated (300 °C for 2 h) dogbone machined specimens. For the XY and the Z building orientation three hatch spacings (0.16 mm, 0.19 mm and 0.22 mm) have been considered for manufacturing the specimens before the machining process. Figure 9a,b plot the ecdf of the differences between the fatigue strengths and the interval plot of the fatigue strength at ${N}_{ref}={2\times 10}^{6}$, respectively, for the XY building direction; whereas Fig. 9c,d plot the ecdf of the differences between the fatigue strengths and the interval plot of the fatigue strength at ${N}_{ref}={2\times 10}^{6}$, respectively, for the Z building direction. Figure 9e shows the interval plot of the fatigue strengths for the investigated hatch spacings and for the XY and the Z building directions, to visualize possible interaction between the investigated factors. In Fig. 9a–d the notations XY16 (Z16), XY19 (Z19), XY22 (Z22) indicate the specimens built in XY (Z) direction with 0.16 mm, 0.19 mm and 0.22 mm hatch spacing, respectively. In Fig. 9e, H16, H19, and H22 refer to specimens built with 0.16 mm, 0.19 mm and 0.22 mm hatch spacing, respectively.

According to Fig. 9a–d, the hatch spacing influences the fatigue strength at the investigated number of cycles, since the median fatigue strength decreases as the hatch spacing increases, in agreement with the trend found in Ref.^12^. Moreover, the difference is not statistically significant by considering the fatigue strength of specimens manufactured with 0.16–0.19 mm and 0.19–0.22 mm hatch distances for both building orientations and for a 5% significance level. On the other hand, the interval plots do not overlap for the fatigue strengths obtained by testing specimens with 0.16 and 0.22 mm hatch spacings and the zero difference is significantly below the difference corresponding to the 5% significance level, for both building orientations. This suggests that the hatch spacing factor significantly affects the fatigue response, even if the scatter of the fatigue response is large, in agreement with Ref.^12^. However, the difference becomes statistically significant only if the hatch spacing difference is large, i.e., above 0.06 mm. Even for this material, the interval plots estimated with the deterministic approach in Ref.^12^ are smaller than those estimated with the methodology developed in the present paper, with the median fatigue strength, on the other hand, being very close.

According to Fig. 9e, the building orientation and the hatch spacing interacts: indeed, for the 0.16 mm hatch spacing the specimens built with the XY building direction are characterized by larger fatigue strength, whereas no difference is found for the 0.22 mm hatch spacing. A similar result has been found in Ref.^12^, where building orientation, hatch spacing and their interactions have been found to be statistically significant factors.

### Maraging steel

**Figure 10:**
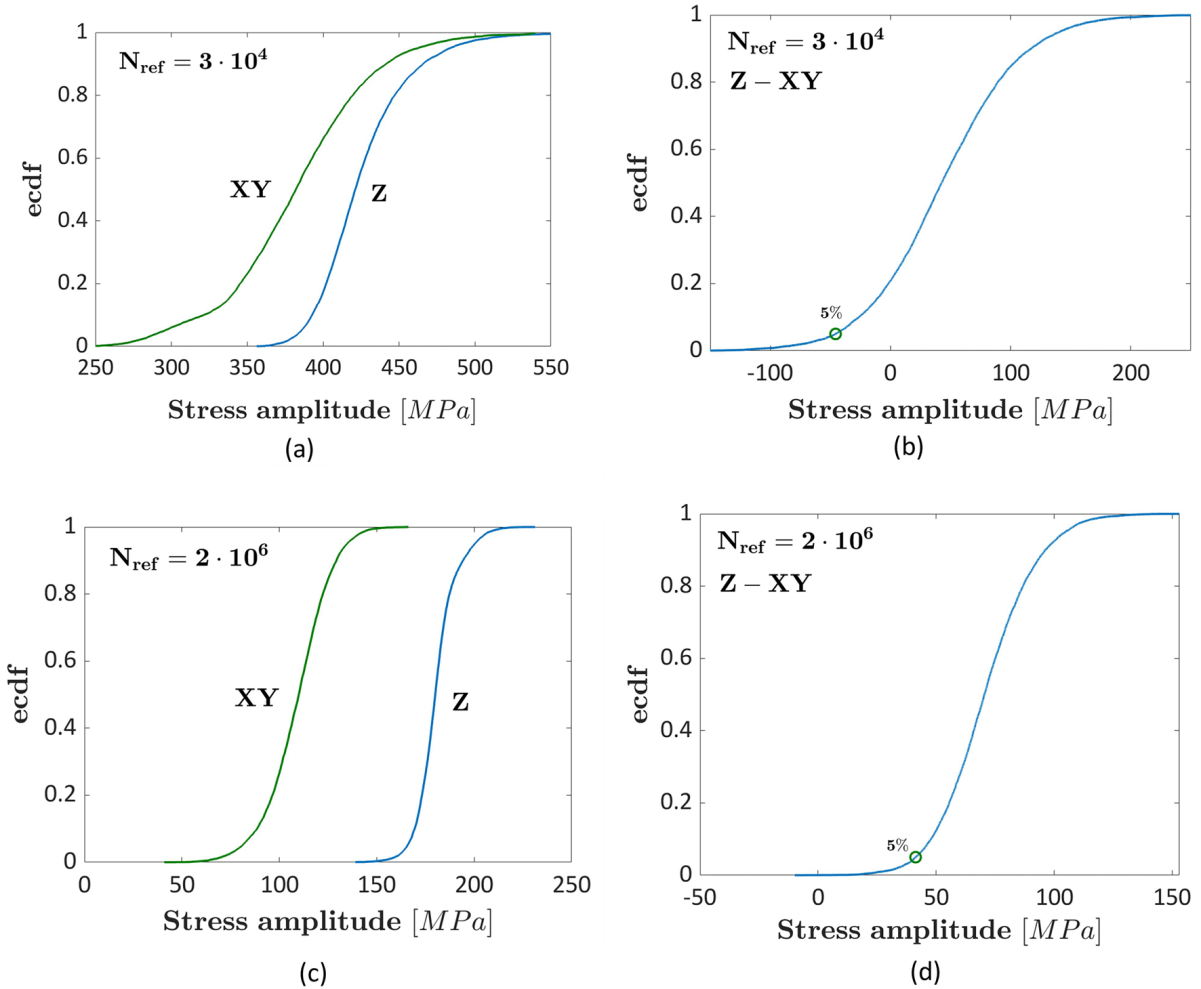
Experimental results in Ref.^41^ on maraging steel specimens: (**a**) ecdf of the fatigue strengths at ${N}_{ref}={3\times 10}^{4}$; (**b**) ecdf of the difference between the XY and the Z building orientation fatigue strength at ${N}_{ref}={3\times 10}^{4}$ cycles; (**c**) ecdf of the fatigue strengths at ${N}_{ref}={2\times 10}^{6}$; (**d**) ecdf of the difference between the XY and the Z building orientation fatigue strength at ${N}_{ref}={2\times 10}^{6}$ cycles, respectively.

Finally, the model has been validated on the experimental results obtained by testing maraging steel specimens^41^. Fully reversed axial fatigue tests have been carried out in Ref.^41^ on polished specimens built in XY and Z directions. The influence of the building orientation and its variation with the fatigue life has been investigated, by analyzing and comparing the fatigue strengths at ${N}_{ref}={3\times 10}^{4}$ cycles and at ${N}_{ref}={2\times 10}^{6}$ cycles. Figure 10a,b plot the ecdfs of the fatigue strengths and the ecdf of the difference between the fatigue strengths at ${N}_{ref}={3\times 10}^{4}$ cycles, respectively. Figure 10c,d plot the ecdfs of the fatigue strengths and the ecdf of the difference between the fatigue strengths at ${N}_{ref}={2\times 10}^{6}$ cycles, respectively.

According to Fig. 10a,c, the median fatigue strength is close to that estimated in the original paper (about 400 MPa at $3\times {10}^{4}$ cycles and 100 MPa at $2\times {10}^{6}$ cycles for the XY building orientation, and about 420 MPa at $3\times {10}^{4}$ cycles and 180 MPa at $2\times {10}^{6}$ cycles for the Z building orientation). According to Fig. 10a, the fatigue strength obtained by testing specimens built in the Z direction is larger, even if with limited differences, as confirmed by Fig. 10b. Indeed, the zero value is significantly above the 5% significance level, thus suggesting that the difference is not statistically significant for failures occurring at ${N}_{ref}={3\times 10}^{4}$ number of cycles. On the other hand, by analysing Fig. 10c,d, the difference becomes statistically significant at ${N}_{ref}={2\times 10}^{6}$ cycles. This result agrees with that found in Ref.^12^ and confirms that the influence of the building orientation increases with the number of cycles to failure for the investigated maraging steel.

## Discussions

In "Methods", a methodology for the analysis of the fatigue results obtained by testing specimens produced through AM processes has been developed. The proposed procedure improves the methodology developed by the Authors in Ref.^12^ and based on the idea of shifting the experimental failures at a reference number of cycles to failure, ${N}_{ref}$. Indeed, the large scatter of AM fatigue data in the S–N plot, together with the limited number of experimental data commonly available, makes it difficult to analyze the fatigue response and to compare, for example, datasets obtained by varying an investigated factor. On the other hand, the Authors, in Ref.^12^, have proven and validated the effectiveness of gathering the experimental failures at ${N}_{ref}$. However, as highlighted in "Methods", one weakness of the approach in Ref.^12^ is that it does not account for the uncertainty associated with the fatigue strength at ${N}_{ref}$. This weakness has been overcome in the present paper, with a novel procedure based on the estimation of the Profile Likelihood function for modelling the experimental variability and assessing the confidence interval within which the fatigue strength at ${N}_{ref}$ may fall, rather than considering a deterministic fatigue strength at ${N}_{ref}$. With this procedure, the influence of the dataset size is also accounted for, with the Profile Likelihood function range increasing or decreasing depending on the number of available data. The strengths and weaknesses of this new approach, compared to those of the methodology in Ref.^12^, are further discussed in this Section.

The original procedure developed in Ref.^12^ is based on a stress-life relationship model which accounts also for the influence of defects on the fatigue response. The so-called “marginal P–S–N curves” are considered in the duplex P–S–N curves. This is a strength of the model in Ref.^12^, since defects contribute to the large experimental scatter. For each experimental failure, together with the applied stress amplitude and the number of cycles to failure, the defect size at the origin of the fatigue failure is thus required as a necessary input. However, the defect size may not be reliably identifiable, especially if the fatigue failure originates from the specimen surface. Moreover, the assessment of the defects at the origin of the fatigue failures requires detailed analyses of the fracture surfaces, which cannot be carried out, if, for example, a Scanning Electron Microscope (SEM) is not available. On the other hand, the procedure developed in the present paper does not require the assessment of the critical defect and can be reliably applied even for fatigue failures originating from the specimen surface, as proven in "Ti6Al4V literature data: influence of AM process and post-treatments in the high cycle fatigue regime". The influence of defects, even if not explicitly accounted for in the present model, is, on the other hand, modelled indirectly by considering the uncertainty associated with the fatigue strengths at ${N}_{ref}$, since defects are the main responsible for the large scatter and uncertainty of experimental data on the S–N plot.

The stress-life relationship considered in Ref.^12^ models the fatigue life up to the VHCF life range with a duplex trend, whereas in the present work a model with a monotonic decreasing trend and a fatigue limit is considered. However, for AM components, experimental data are mainly available in the Low Cycle Fatigue (LCF)-HCF life range or in the VHCF range. Accordingly, the model proposed in the present paper can be reliably employed for most of the available AM datasets. It must be also noted that the methodology considered in the present paper can be further developed by considering a duplex trend in the S–N plot, provided that a large number of experimental data up to the VHCF life range is available.

The proposed procedure has, moreover, been implemented by estimating the Profile Likelihood function for modelling the variability of the fatigue strengths ${N}_{ref}$. According to Ref.^42^, indeed, confidence intervals based on the Profile Likelihood function provides better coverage probabilities than traditional Normal-based confidence intervals and are indicated when the number of available data is limited, as for results of fatigue tests on AM components. On the other hand, the estimation of the Profile Likelihood function is complex, requiring multiple optimizations, but it can be easily managed by numeric software, following the straightforward procedure described in the paper.

The developed procedure has been proven to be more conservative than the one developed in Ref.^12^. Indeed, the intervals for the fatigue strengths at ${N}_{ref}$ have been found to be wider. This can be expected and can be explained by considering that, rather than a deterministic fatigue strength at ${N}_{ref}$, an interval accounting for the uncertainty in the estimation of the quantile fatigue strength and for the dataset numerosity has been considered.

It must be noted that the variability of the fatigue response in the S–N plot can be also attributed to the variation of the failure mode and the failure mechanisms with the applied load and life range. In this case, the experimental variability is expected to increase, with a significant change in the trend of the experimental data in the S–N plot. Accordingly, the linear monotonic decreasing trend considered in the present work does not provide the best fitting and more complex statistical models should be considered for the initial fitting of the experimental results, e.g., a model capable to describe a duplex trend in the S–N plot^43^. Indeed, if the experimental data are not fitted with the appropriate statistical model, the proposed approach may provide misleading results, with the source of the experimental variability not reliably modeled. The proposed approach is capable of modelling the occurrence of different failure modes, provided that the investigated failure mechanisms significantly affect the fatigue response with a changing trend of the experimental data in the S–N plot. Accordingly, a statistical model capable of following the experimental trends in the S–N plot should be considered for the initial fitting, but the number of experimental data should be large enough to ensure a reliable estimate of the unknown parameters.

The results of the analysis carried out with the proposed methodology should be carefully interpreted. For example, according to Figs. 5 (influence of manufacturing process) and 9 (influence of hatch spacing), the median fatigue at ${N}_{ref}$ tends to decrease with the investigated factors. The ecdf of the difference or the analysis of the interval plot have shown that, in a statistical framework, the difference cannot be considered significant for a significance level above 5%, due to the large variability of the fatigue response. It must be noted that this does not necessarily mean that the investigated factor has no effect, but, on the other hand, that the available data may not be sufficient to prove its effect with high reliability and that more data may be necessary to achieve the required significance level. The influence of the amount of data in the shifted region has been shown in Figs. 5 and 6, where different conclusions on the effect of the manufacturing process have been drawn depending on the considered life range. Accordingly, the proposed methodology can also be employed to verify in which life region more data are required to draw statistically significant conclusions supported by the data. For limited amounts of data in a specific life region, the estimated interval for the fatigue strength at ${N}_{ref}$ tends to be very large, making it difficult to draw conclusions with high confidence levels and concealing the influence of the investigated factor.

It must be noted that the fatigue response of AM parts is strongly influenced by the microstructure and the defect population, with both also interacting and influencing the crack nucleation process and, consequently, the fatigue life. According to Refs.^29,30^, the defect size affects the slope of the S–N curves. The proposed approach is statistical-phenomenological, and involves, as a first step, the selection of the fatigue life model that allows for the best fitting of the experimental data (monotonic decreasing or monotonic decreasing with a final asymptote). The trend of the estimated S–N curve varies depending on the defect population and part microstructure, with the estimated constant coefficients accounting for the influence of all factors affecting the fatigue life. Accordingly, the variation of the trend and the slope of the fatigue life with the microstructure or the defect population is reliably modeled by the proposed method, according to the literature^29,30^. On the other hand, the proposed approach cannot discriminate which factor mostly affects the fatigue response, since all factors influencing the crack nucleation process are included and “hidden” in the estimated constant coefficients.

To conclude, the proposed methodology has proven its effectiveness and its capability of accounting for and modelling the variability of AM fatigue results, allowing to draw reliable statistically significant conclusions and providing important information for the interpretation of the results in a statistical framework. Although it has been developed for AM specimens, it can be also reliably employed for traditionally built materials, thanks to its flexibility and adaptability.

## Conclusions

In the present paper, a methodology for modelling the experimental scatter of the fatigue response of components produced through Additive Manufacturing (AM) processes is proposed. The methodology is based on shifting the experimental failures at a reference number of cycles, ${N}_{ref}$, thus allowing for subsequent analyses with reliable statistical methodologies, even though the data are collected at different number of cycles. Instead of a deterministic fatigue strength at ${N}_{ref}$, an interval is estimated by exploiting the Profile Likelihood function properties. The following conclusions can be drawn:

1. The proposed approach can be reliably applied even if the defect at the origin of the fatigue failure is not available. The influence of defects on the fatigue response is indirectly accounted for by considering the confidence interval, estimated with the Profile Likelihood function, for the fatigue strength at ${N}_{ref}$.
2. The approach is particularly effective when datasets obtained by varying an investigated factor are compared. With the data shifted at ${N}_{ref}$, the empirical cumulative distribution function of the difference between the fatigue strengths can be assessed or the interval plots can be compared. The validity of this methodology has been proven by investigating the effect of manufacturing processes, building orientation and hatch spacing in literature works.
3. The method has been validated by considering literature datasets on AM specimens. It provides more conservative estimations of the fatigue strength with respect to those of the other available literature models, since the range of uncertainty of the fatigue strength at ${N}_{ref}$ is also modelled.
4. The results of the analyses carried out with the present approach should be correctly interpreted, to assess if the influence of an investigated factor is relevant, e.g., by considering the median fatigue strength trend, but concealed by the large experimental variability due to the limited amount of data in that specific life region. In these cases, by analysing the fatigue strengths at different ${N}_{ref}$, it is possible to investigate the source of variability, as proven in the paper.

To conclude, the proposed approach effectively addresses one of the main open issues concerning the structural integrity of AM parts, i.e., the large variability of the fatigue response. However, thanks to its flexibility, it can be also adapted and employed for parts produced with traditional manufacturing processes.

## Data Availability

In the paper, the data used for the validation of the proposed statistical method have been retrieved in the literature from published papers. These data are therefore already available in the literature. New data have not been generated in the paper.
